# Artificial Intelligence in Temporomandibular Joint Disorders: An Umbrella Review

**DOI:** 10.1002/cre2.70115

**Published:** 2025-03-11

**Authors:** Vini Mehta, Snehasish Tripathy, Toufiq Noor, Ankita Mathur

**Affiliations:** ^1^ Faculty of Dentistry University of Ibn al‐Nafis for Medical Sciences San'a Yemen; ^2^ Department of Dental Research Cell Dr. D. Y. Patil Dental College and Hospital, Dr. D. Y. Patil Vidyapeeth Pune India

**Keywords:** artificial Intelligence, machine learning, temporomandibular joint disorders

## Abstract

**Objectives:**

Given the complexity of temporomandibular joint disorders (TMDs) and their overlapping symptoms with other conditions, an accurate diagnosis necessitates a thorough examination, which can be time‐consuming and resource‐intensive. Consequently, innovative diagnostic tools are required to increase TMD diagnosis efficiency and precision. Therefore, the purpose of this umbrella review was to examine the existing evidence about the usefulness of artificial intelligence (AI) in TMD diagnosis.

**Material and Methods:**

A comprehensive search of the literature was performed from inception to November 30, 2024, in PubMed‐MEDLINE, Embase, and Scopus databases. This review evaluated systematic reviews (SRs) and meta‐analyses (MAs) that reported TMD patients/datasets, any AI model as intervention, no treatment, placebo as comparator and accuracy, sensitivity, specificity, or predictive value of AI models as outcome. The extracted data were complemented with narrative synthesis.

**Results:**

Out of 1497 search results, this umbrella review included five studies. One of the five articles was an SR while the other four were SRMAs. Three studies focused on patients with temporomandibular joint (TMJ) problems as a group, whereas two were specific to temporomandibular joint osteoarthritis (TMJOA). The included studies reported the use of imaging datasets as samples, including cone‐beam computed tomography (CBCT), magnetic resonance imaging (MRI), and panoramic radiography. The studies reported an accuracy level ranging from 0.59 to 1. Four studies reported sensitivity levels ranging from 0.76 to 0.80. Four studies reported specificity values ranging from 0.63 to 0.95 for TMJ conditions. However, only one study provided the area under the curve (AUC) in the diagnosis of TMDs.

**Conclusions:**

AI has the ability to provide faster, more accurate, sensitive, and objective diagnosis of TMJ condition. However, the performance is determined on the AI models and datasets used. Therefore, before implementing AI models in clinical practice, it is essential for researchers to extensively refine and evaluate the AI application.

## 1 Introduction

Temporomandibular disorders (TMDs) are painful and debilitating craniofacial conditions that affect the temporomandibular joints (TMJs) and masticatory muscles, leading to restricted range of motion, joint noise, and distorted mouth opening due to joint dysregulation. Worldwide, 34% of individuals, the majority of whom are between the ages of 18 and 60, have reported experiencing symptoms of TMJ (Zieliński et al. 2024). TMJ disorders have a complex and multifaceted etiology that has been linked to comorbidities such as heart disease, osteoarthritis, hearing loss, sinusitis, and thyroid dysfunction. TMJ issues have long‐term consequences for both individuals and society. TMD can lead to lasting complications, such as malocclusion or facial deformities. Additionally, it can contribute to mental health challenges. A recent systematic study found that 43.0% of TMD patients had depression and 60.0% had somatization (Felin et al. 2024). TMJ dysfunction also causes productivity loss along with substantial medical expenses, negatively affecting the economy (Yost et al. 2020). Therefore, early diagnosis, classification, and management of TMJ are essential for enhancing treatment effectiveness, alleviating symptoms, implementing preventive strategies, maintaining joint mobility, and optimizing healthcare resource utilization. Early detection and intervention may drastically enhance patient outcomes and quality of life, potentially decreasing the need for more invasive and expensive therapies (Xu, Chen, et al. 2023).

The current criteria for diagnosing TMJ disorders include a comprehensive medical history to rule out an underlying cause of the disorder and a TMJ joint assessment by a dentist or oral maxillofacial specialist to determine the extent of movement, joint sounds, tenderness, and any signs of inflammation or swelling. Furthermore, diagnostic imaging techniques such as X‐rays, computed tomography (CT), and medical resonance imaging are used to get comprehensive views of the TMJ and its neighboring tissues (Xu, Chen, et al. 2023; Ozsari et al. 2023). In addition, the diagnostic criteria for TMDs (DC‐TMD), developed in the 1990s, remains the most extensively used diagnostic criteria to date. However, it has drawbacks such as limited diagnostic accuracy, sensitivity, and specificity (Jha et al. 2022). As a result, given the complexity of TMDs and their overlapping symptoms with other conditions, an accurate diagnosis necessitates a thorough examination, which can be time‐consuming and resource‐intensive. Consequently, innovative diagnostic tools are required to increase TMD diagnosis efficiency and precision.

Artificial intelligence (AI) has advanced rapidly in recent years. Its subfields, including machine learning (ML) and deep learning, have garnered significant attention for their ability to autonomously learn from diverse datasets, such as images, texts, videos, and more (Xu, Chen, et al. 2023). AI models have also demonstrated superior predicted accuracy, speed, and efficiency in medical diagnostic procedures as compared to traditional approaches (Abd‐alrazaq et al. 2022; Zha et al. 2024; Huang et al. 2024). These AI algorithms are capable of analyzing medical images such as traditional X‐rays, CT scans, magnetic resonance imaging (MRI), and ultrasound, allowing physicians to identify and diagnose diseases more precisely and faster. Its application has been observed across various health domains, including dentistry, neuropathies (Yetiş et al. 2024), and so on. Several systematic reviews (SRs) have documented the use of AI in diagnosing TMD. The accuracy of AI models varies depending on factors such as the model used, the participant population, data inputs, and performance metrics. Therefore, an umbrella review (UR) was needed to synthesize the existing body of knowledge, assess the quality of current studies, and identify gaps in the research. The aim of this UR was to evaluate the evidence on the effectiveness of AI in diagnosing TMD. The results will inform future research and support the integration of AI into standard TMD diagnostic practices.

## 2 Methods

### 2.1 Review Registration

This UR was carried out in full compliance with the predefined protocol registered in the international prospective register of SRs, PROSPERO database. We followed the methodology outlined by Aromataris et al. (2020) and PRIOR guidelines (Pollock et al. 2019) for conducting the UR, which includes key components such as a systematic search, eligibility criteria, screening process, data extraction, critical assessment, and the presentation of both quantitative and qualitative systematic review findings in a clear and comprehensible manner. The UR checklist has been provided in Supporting Information S1: Table S1.

#### 2.1.1 Search Strategy

A comprehensive literature search strategy was developed to identify SRs that examined AI models in TMJ problem diagnosis and treatment. The search was completed online on November 30, 2024, which covered systematic reviews (SRs) with or without meta‐analysis (MA), regardless of language or date restrictions. The search utilized three databases: PubMed‐MEDLINE, Embase, and Scopus. The search approach was developed initially for MEDLINE (PubMed). Each search concept entailed Medical Subject Headings (MeSH) and synonymous key terms, which were joined using Boolean operators. The search was then customized for each database to follow the particular database search standards. The supplementary material (Supporting Information S1: Table S2) includes detailed search algorithms for all databases.

### 2.2 Eligibility Criteria

The inclusion and exclusion criteria for this UR were formed based on the PICOS (P = Population, I = Intervention, C = Comparator, O = Outcome, S = Study) framework where

P*opulation (P)* = temporomandibular joint disorders patients/datasets of TMJ patients,

I*ntervention (I)* = any artificial intelligence model,

C*omparators (C)* = no treatment, placebo, or other conventional interventions,

O*utcome (O)* = accuracy, sensitivity, specificity, or predictive value of AI models in the diagnosis, treatment, or prediction of TMJ disorders/treatments,

The *study design (S)* included only SRs or SRMAs that reported the outcomes of interest.

We excluded the studies, which were literature reviews, narrative reviews, rapid reviews, URs, or other primary research designs.

### 2.3 Selection Process

The studies were selected in two key stages: title/abstract screening and full‐text screening. First, the search results for each database were obtained in the research information systems (RIS) format. These RIS files were subsequently imported into Rayyan, an online systematic review application, to aid in the initial filtering of the search results. Two authors independently and blindly reviewed the titles and abstracts. Any discrepancies were resolved through discussion with a third reviewer to reach a unanimous decision. The full text of all potentially eligible studies was then downloaded for further screening. The same two authors assessed the full‐text articles according to the inclusion criteria, with any disagreements addressed through consultation with the third author.

### 2.4 Data Extraction

To ensure uniformity in extraction, the authors developed and evaluated a data extraction form on two randomly selected papers for this UR. A pilot test was used to modify the template. Any disagreement in data extraction was resolved after consultation with a third author. The extracted information included general study characteristics (authors, year of publication, and country of origin), protocol registration, language, time limit, PICOS structure, search details (databases, number of studies detected and included), critical appraisal, and review of key findings (AI model, type of data, sample size, key outcomes). Overlapping studies were identified in the included reviews using a citation matrix (Supporting Information S1: Figure S1).

### 2.5 Risk of Bias Assessment of the Included SRs

Two reviewers independently assessed the risk of bias in the included reviews using the AMSTAR‐2 critical appraisal checklist (Shea et al. 2017). The checklist included a total of 16 questions divided into critical and noncritical domains. Each question on the checklist can be assigned responses such as Yes, No, or Partial Yes. Based on the fulfillment of important or noncritical domains, the total confidence in the included review is divided into four main categories: high confidence (no or one noncritical weakness), medium confidence (> 1 weakness but no critical weakness), low confidence (one critical weakness with or without noncritical weakness), and critically low confidence (> 1 critical weakness with or without noncritical weakness). Any differences were resolved through conversation with a third author.

### 2.6 Data Curation and Synthesis

The first author evaluated and summarized the evidence and data, which was then validated by the second author. This information was then compiled in a tabular format and presented descriptively.

## 3 Results

**Figure 1:**
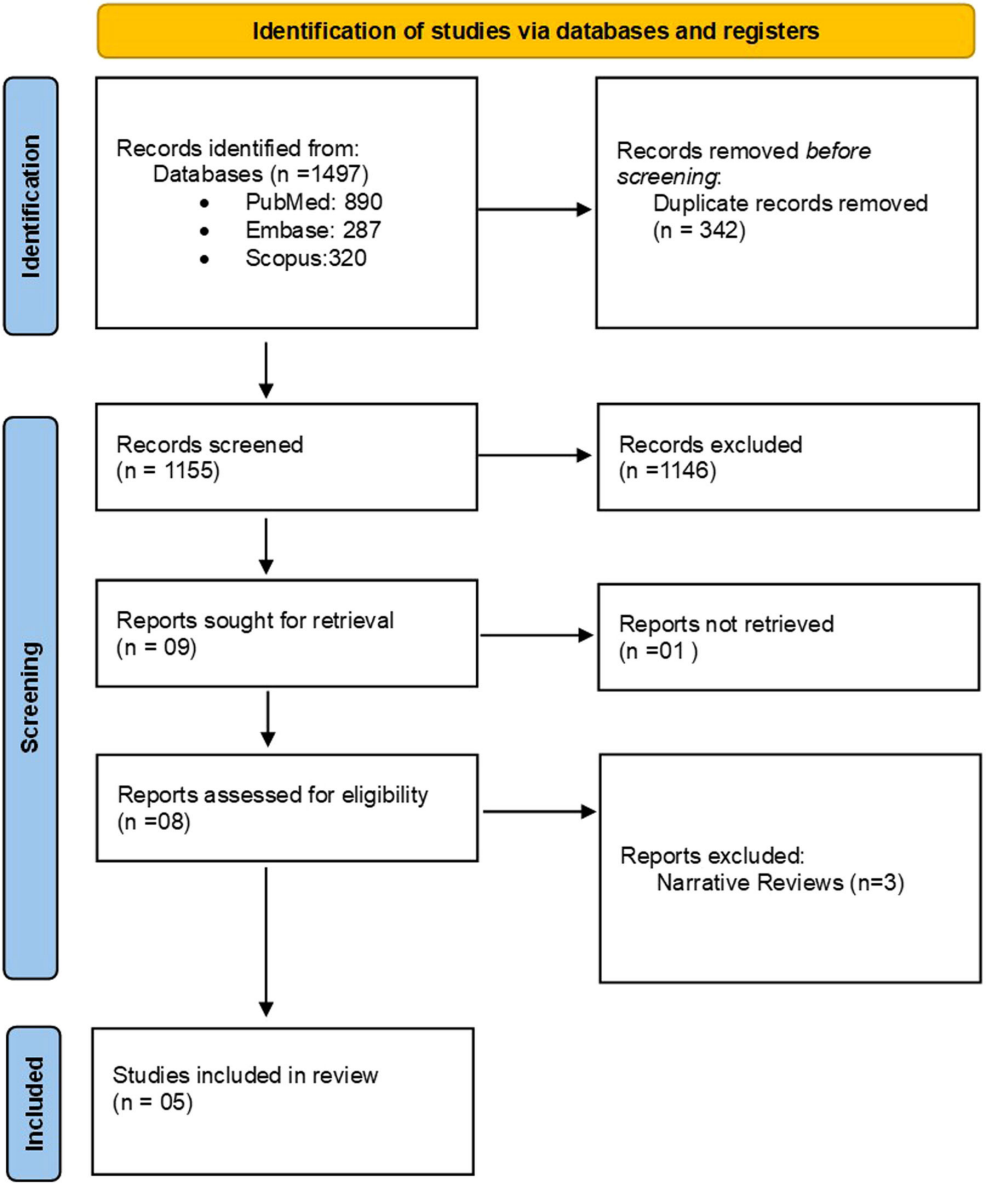
PRISMA flowchart depicting the study selection process (PRISMA 2020).

The database search identified 1497 studies, of which 342 were duplicates and removed. This left 1155 studies for eligibility assessment. Following title and abstract screening, 1146 studies were deemed ineligible, leaving nine studies for full‐text retrieval. After a thorough evaluation of the full‐text articles, three studies were excluded as they were narrative reviews. Consequently, this UR included five studies (Figure 1).

### 3.1 Study Characteristics

The included reviews were published in the last 2 years (2022–2024). One of the five articles considered was an SR (Farook and Dudley 2023), while the other four were SRMAs (Xu, Chen, et al. 2023; Jha et al. 2022; Almășan et al. 2023; Zhang et al. 2024). Three studies focused on patients with TMJ problems as a group, whereas two were specific to temporomandibular joint osteoarthritis (TMJOA). Four research used an AI model, while one used deep learning and automation for TMJ diagnosis. The comparator in the studies ranges from none to clinical diagnosis based on medical diagnostic images/patient data. The outcomes comprised AI performance indicators such as accuracy, sensitivity, specificity, and area under the curve for TMJ subtype diagnosis. The detailed PICO format of the included studies is provided in Table 1.

**Table 1:** PICO elements of included studies.

Author and year of publication	Population	Intervention	Comparator	Outcome
Almășan et al. (2023)	Patients with TMJ osteoarthritis	AI as a diagnosis method	None/Human	Sensitivity and specificity
Jha et al. (2022)	Patients with TMDs	Type of data and algorithm for an AI‐based automated diagnostic model	Use of medical diagnostic images (CBCT, MRI, panoramic radiographs) and patient records	Performance of AI algorithms assessed using diagnostic accuracy
Xu, Chen, et al. (2023)	Patients with TMJOA	AI models applied to radiographic imaging for TMJOA detection	NA	Sensitivity, specificity, area‐under‐the‐curve value
Zhang et al. (2024)	TMD patients	Any diagnostic test based on machine learning (including deep learning)	Clinical diagnosis by physicians	Sensitivity and specificity
Farook and Dudley (2023)	Patients with TMJ disorders, including TMJ osteoarthritis, disc disorders, and trauma	Automation and deep learning applied to TMJ radiomics using MRI, CBCT, panoramic radiographs, and thermographic imaging	Practitioners' diagnostic assessments or manually annotated datasets	Disc disorders, trauma, and temporomandibular joint pathologies.

Abbreviations: AI, artificial intelligence; CBCT, cone beam computed tomography; MRI, magnetic resonance imaging; TMD, temporomandibular disorders; TMJ, temporomandibular joint; TMJOA, temporomandibular joint osteoarthritis.

The reviewers of the included studies were affiliated with institutions from South Korea (*n* = 1), Australia (*n* = 1), Romania (*n* = 1), and China (*n* = 2). Four research indicated registering their study procedures in the open science framework (OSF) [14] and PROSPERO (*N* = 3) databases (Xu, Chen, et al. 2023; Farook and Dudley 2023; Zhang et al. 2024), whereas one study did not mention of a priori protocol registration (Jha et al). Two studies (Farook and Dudley 2023; Zhang et al. 2024) used PRISMA‐DTA guidelines for their reviews, while three followed PRISMA guidelines. Three of the included publications utilized the QUDAS‐2 tool to assess the quality of the primary studies (Xu, Chen, et al. 2023; Jha et al. 2022; Zhang et al. 2024), one used QUADAS 2 and the MI‐CLAIM checklist (Almășan et al. 2023), and one used the MI‐CLAIM checklist and the Cochrane GRADE tool (Farook and Dudley 2023). In terms of the search approach, one study placed a time limit beginning in 2010, but the other four studies searched from the database's inception. Furthermore, three of the studies applied an English‐language filter to their search (Xu, Chen, et al. 2023; Jha et al. 2022; Farook and Dudley 2023) but the other two did not (Table 2).

**Table 2:** Study characteristics.

Author and year of publication	Country of origin	Protocol registration	Guidelines followed	Search duration	Language restriction	Critical appraisal tool
Almășan et al. (2023)	Romania	Open Science Framework	PRISMA	Inception to May 28, 2022	None specified	QUADAS‐2 and MI‐CLAIM checklist
Jha et al. (2022)	South Korea	Not registered	PRISMA	Inception to June 30, 2022	English	QUADAS‐2
Xu, Chen, et al. (2023)	China	PROSPERO	PRISMA	January 2010 to January 2023	English	QUADAS‐2
Zhang et al. (2024)	China	PROSPERO	PRISMA‐DTA	Inception to up to July 19, 2023	None specified	QUADAS‐2
Farook and Dudley (2023)	Australia	PROSPERO	PRISMA‐DTA	Inception to October 01, 2022	English	MI‐CLAIM checklist and Cochrane's GRADE approach

Abbreviations: GRADE, grading of recommendations, assessment, development, and evaluation; MI‐CLAIM, minimal information for complex low‐risk artificial intelligence models; PRISMA, preferred reporting items for systematic reviews and meta‐analyses; PRISMA‐DTA, preferred reporting items for systematic reviews and meta‐analyses of diagnostic test accuracy studies; PROSPERO, prospective register of systematic reviews; QUADAS‐2, quality assessment of diagnostic accuracy studies‐2.

The included studies conducted a thorough and rigorous search across many databases, with PubMed, Web of Science, Embase, and Scopus being the most commonly used. The number of databases searched varied from three to twelve. Zhang et al. (2024) conducted a more comprehensive search, including searches in databases, preprint servers, and trial platforms. The number of results retrieved varied from 203 to 1923, with an average of approximately 901 studies. The total number of studies included ranged from 6 to 28, with an average of 16 studies. The included studies reported using a variety of imaging datasets as samples, including CBCT, MRI, and panoramic radiography. Only three research mentioned the data sets' sample sizes, which ranged from 28 to 10,077 TMJ images (Xu, Chen, et al. 2023; Zhang et al. 2024) (Table 3).

**Table 3:** Database search and key findings of included studies.

Author and year of publication	Database searched	Total studies found	Total included studies	Sample population	Sample size	Key findings
Almășan et al. (2023)	Pubmed, Embase, Scopus, Web of Science, LILACS, ProQuest, SpringerLink	203	7	Structured (clinical and biomolecular) and unstructured (imaging) TMJ images	10,077 TMJ images analyzed, with 5520 images in meta‐analysis	Accuracy: higher accuracy noted for fine‐tuned models (e.g., XGBoost + LightGBM achieved 82.3% accuracy) Pooled sensitivity of 0.76 (95% CI 0.35–0.95) ResNet classifications Pooled specificity of 0.79 (95% CI 0.75–0.83)
Jha et al. (2022)	PubMed, Embase, Web of Science	1923	17	Medical imaging data (CBCT, MRI, radiographs)	Not mentioned	The diagnostic accuracy was 0.69–1.00, and the pooled accuracy was 0.91
Xu, Chen, et al. (2023)	Pubmed, Web of Science, Scopus, Embase	513	6	Imaging data (MRI, CBCT, and OPG)	523 images with TMJOA and 734 images from controls	Pooled accuracy of 0.92 Pooled sensitivity of 0.80 (95% CI: 0.67–0.89) Pooled specificity of 0.90 (95% CI: 0.87–0.92); AUC was 0.92 (95% CI: 0.89–0.94)
Zhang et al. (2024)	Europe PMC; Embase via Ovid, EBM Reviews via Ovid, Scopus, Web of Science, Information Service in Physics, Electro‐Technology and Computer and Control (Inspec), Korea Citation Index (KCI), (SciELO), WHO Global Index Medicus (GIM), arXiv.org, OSF Preprints, and IEEE Xplore. Two trail platforms: WHO ICTRP, and ClinicalTrials.org.	1660	28 studies (29 reports)	MRI (*n *= 8), panoramic radiographs (*n* = 4), cone‐beam computed tomography (CBCT, *n* = 11), and other image modalities (*n* = 5).	28	Diagnosis of DJD with CBCT using random forest Sensitivity: 0.745 Specificity 0.770: Diagnosis of DJD with CBCT using XGBoost: Sensitivity: 0.765 Specificity 0.766 Diagnosis of DJD with CBCT using LightGBM: Sensitivity: 0.781 Specificity: 0.781
Farook and Dudley (2023)	MEDLINE, EBSCOHost, Scopus, PubMed, Web of Science	208	20	MRI, CBCT, panoramic radiographs, and thermographic imaging	Not mentioned	Accuracy: 0.59–0.92 Sensitivity: 0.54–0.99 Specificity: 0.63–0.95

Abbreviations: CBCT, cone beam computed tomography; CI, confidence interval; DJD: degenerative joint disease; MRI, magnetic resonance imaging; OPG, orthopantomogram; TMJ, temporomandibular joint; TMJOA: temporomandibular joint osteoarthritis.

#### 3.1.1 Summary of AI Classification Models and Their Performance Metrics

The included studies revealed the application of a variety of AI models for TMJ diagnosis such as convoluted neural networks (*n* = 3), artificial neural networks (*n* = 2), deep neural networks (*n* = 3), decision trees (*n* = 2), support vector machines (*n* = 2), K‐nearest neighbors (KNNs) (*n* = 3), and deep learning models such as ResNet, Inception V3, and so on.

Four studies reported the diagnostic accuracy of AI models. The studies reported an accuracy level ranging from 0.59 (Shea et al. 2017) to 1 (Almășan et al. 2023; Talaat et al. 2023). Notably, Almasan et al. noted a high accuracy level with fine‐tuned ML models (e.g., XGBoost + LightGBM) up to 82.3% accuracy. Farook and Dudley (2023) found a sensitivity of 0.63–0.95 utilizing deep learning models.

Four studies reported sensitivity levels ranging from 0.76 to 0.80. Almășan et al. (2023) found a pooled sensitivity of 0.76 for ResNet classifications in TMJ Joint Osteoarthritis Diagnosis E, while Jha et al. (2022) discovered a pooled sensitivity of 0.80 for CNNs and KNNs. Zhang et al. (2024) exhibited 100% sensitivity in identifying degenerative joint conditions using SVM, random forest, logistic regression, and Yolov5. In contrast, Inception V3 exhibited 100% sensitivity in diagnosing disc displacement. Zhang et al. (2024) observed that LightGBM (0.79) had a higher sensitivity in diagnosing degenerative joint conditions using CBCT than XGBoost (0.76) and random forest (0.76). Farook and Dudley (2023) reported a specificity of 0.54–0.99 for TMJ radiomics.

Four studies (Xu, Chen, et al. 2023; Farook and Dudley 2023; Almășan et al. 2023; Zhang et al. 2024) found specificity values ranging from 0.63 to 0.95 for TMJ radiomics. Farook and Dudley (2023) reported a pooled specificity of 0.79 for deep learning models in TMJ Osteoarthritis Diagnosis E, while Farook and Dudley (2023) reported a specificity of 0.54–0.99. Xu et al. found a pooled specificity of 0.80 using CNNs. Zhang et al. (2024) revealed that the ANN model's specificity in disc displacement is 91.8%. On the other hand, LightGBM (0.781) outperformed XGBoost (0.7650) and random forests (0.745) in detecting DJD using CBCT. Only one study reported an area under the curve, with AUC values ranging from 0.89 to 0.54 to 0.89 of CNN and KNN in the diagnosis of TMJ osteoarthritis (Jha et al. 2022).

#### 3.1.2 Quality Appraisal Findings

One study was deemed to be of poor quality, one was of severely low quality, and the remaining three were medium quality. The tool's least met domain was reporting funding for included studies and the list of excluded studies (Table 4).

**Table 4:** Quality appraisal using the AMSTAR 2 (assessment of multiple systematic reviews‐2) checklist of critical and noncritical domains.

Author and year of publication	Q1	Q2	Q3	Q4	Q5	Q6	Q7	Q8	Q9	Q10	Q11	Q12	Q13	Q14	Q15	Q16	Overall rating
Almășan et al. (2023)	Yes	Yes	Yes	Yes	Yes	Yes	Partial Yes	Yes	Yes	No	Yes	Yes	Yes	Yes	No	Yes	Low confidence
Jha et al. (2022)	Yes	No	Yes	Yes	Yes	Yes	Yes	Yes	Yes	No	Yes	Yes	Yes	Yes	No	Yes	Critically low confidence
Xu, Chen, et al. (2023)	Yes	Yes	Yes	Yes	Yes	Yes	Partial Yes	Yes	Yes	No	Yes	Yes	Yes	Yes	Yes	Yes	Medium confidence
Zhang et al. (2024)	Yes	Yes	Yes	Yes	Yes	Yes	Partial Yes	Yes	Yes	Yes	Yes	Yes	Yes	Yes	Yes	Yes	Medium confidence
Farook and Dudley (2023)	Yes	Yes	Yes	Yes	Yes	No	Partial Yes	Yes	Yes	No	NA	NA	Yes	Yes	NA	Yes	Medium confidence

*Note:* The colour is highlighting the adherence of the systematic reviews to quality parameters.

## 4 Discussion

TMJ disorder patients often suffer due to inaccurate or delayed diagnosis due to the complex and multifactorial etiology of the disorders. Moreover, the pain associated with TMJ is obscure because it often spreads to other locations distant from joints or related muscles, thus further increasing the diagnosis complexity. Although the Research Diagnostic Criteria for TMDs (RDC/TMD) is a reliable diagnostic tool, the interpretation of TMD signs is largely subjective. Therefore, there is an urgent need to eliminate this subjectivity to improve the diagnosis of TMJ disorders (Talaat et al. 2023). Thus, this UR presents evidence on the performance of AI models in detecting TMJ diseases by combining data on measures such as accuracy, sensitivity, specificity, and area under the curve, so providing a valuable resource to inform future research. In our comprehensive assessment of AI models for TMJ diagnosis, we included evidence from five SRs with and without MA encompassing 78 primary studies.

The findings demonstrate that AI models have excellent sensitivity, specificity, and accuracy for diagnosing TMJ conditions; nevertheless, their efficiency is significantly dependent on the different AI algorithms and the data set type. Fine‐tuned ML models, such as XGBoost and LightGBM, have higher diagnosis accuracy levels than other AI models. XGBoost has demonstrated great accuracy in the detection of other diseases, such as lung cancer (Guan et al. 2023), Alzheimer's disease (Yi et al. 2023), chronic renal disease (Ogunleye and Wang 2018), depression (Sharma and Verbeke 2020), and so on. XGBoost, also called extreme gradient boosting (XGBoost), is a gradient boosting decision tree‐based algorithm that is well‐known for its great efficiency, adaptability, and portability. It is utilized in data extraction, recommendation systems, and other domains (Song et al. 2022). In addition, it is relatively cheaper, simpler, and easier to interpret than neural networks. Furthermore, it outperforms single ML models such as logistic regression, support vector machine, and decision tree (Guan et al. 2023). Similarly, LightGBM is an efficient gradient boosting method‐based algorithm. The key advantage is that it significantly accelerates the training process, leading to better‐performing models. It is based on decision tree algorithms and performs well in classification and regression tasks, surpassing other predictive models. It can optimize decision support systems by determining the number, depth, and leaf nodes of decision trees. LightGBM has been extensively used to diagnose illnesses such as diabetes (Rufo et al. 2021), coronary artery disease (Omotehinwa et al. 2024; Yang et al. 2023), and Parkinson's disease (Dhruva Kumar et al. 2022). Overall, both models—XGBoost and LightGBM—perform better than neural networks in various structured data tasks because of their strong feature selection, lower prediction time, integrated over‐fitting preventive features, small‐to‐medium data set compatibility, and ability to handle tabular data such as effectively such as patient records (Huang and Chen 2021). While neural networks are excellent at tasks involving unstructured data, they frequently fall short of gradient boosting models in structured datasets.

ResNet, Inception V3, and machine LightGBM have shown improved sensitivity and specificity in diagnosing specific TMJ problems. This can be attributed to their distinct advantage in feature extraction and categorization. ResNet (Residual networks) established layer‐level linkage, which eliminates the gradient disappearance problem in deep networks, allowing it to effectively and rapidly assimilate detailed patterns in complex datasets such as MRI or CBCT scans. Its deep architecture allows it to collect fine and broad structural information of images, improving its ability to detect small anomalies (Xu, Fu, et al. 2023; Roy et al. 2021; Al‐Haija and Adebanjo 2020). Similarly, Inception V3 is made up of multilayer convolutional neural networks with varying filter sizes, allowing it to process intricate features at various levels of detail and thus identify invisible trends or small anomalies in TMJ imaging data that the human eye may miss (Guan et al. 2019). As a result, both deep learning models have demonstrated great sensitivity and specificity in a variety of conditions, including breast cancer, fundus disease (Pan et al. 2023), and Alzheimer's disease (Roy et al. 2021). Collectively, these models offer a major improvement in the accuracy and efficiency of AI‐powered healthcare solutions.

While accuracy, sensitivity, and specificity were reported in all investigations, only one study reported area under curve (AUC) values (0.54–0.89). AUC is a statistic that compares the true positive rate to false positives at various threshold values, with greater AUC indicating better performance (Bradley et al. 2019). While the findings of a single study indicate greater performance, the small number of studies on the topic implies an inadequate assessment of predictive performance. This indicates that although AI models show potential, further research and validation are needed to ensure their reliability, generalizability, and successful integration into clinical practice.

### 4.1 Strengths and Limitations

This UR has several strengths. We adhered to PRISMA and PRIOR guidelines for conducting a systematic search and rigorous analysis of the included studies. Additionally, all assessment phases were carried out by two reviewers, with consistent results. However, there are some limitations to this study. The main limitation is the inability to statistically synthesize the data due to variations in AI models, datasets, and analysis methods across the included studies. Consequently, statistical tests could not be applied to evaluate publication bias within the selected studies. Furthermore, many of the included reviews did not meet SRs guidelines, such as failure to report protocol registration, the funding sources of included papers, or a list of excluded studies. This reduces transparency and raises concerns about potential selection bias. Moreover, most of the reviews did not provide the necessary data to assess classifier performance, such as true positives, true negatives, and false positives. Another limitation is that, although we identified overlapping research, we did not exclude these studies. As a result, the performance ranges presented in our review may include some repetitions. We also did not exclude reviews based on quality, as the majority of the collected reviews were rated as low to moderate. Additionally, although we identified overlapping studies across the included SRs and meta‐analyses using a citation matrix, we did not adjust the findings to account for potential duplication of primary studies. As a result, some outcomes may reflect redundant data, which could influence the interpretation of the pooled evidence. Future analyses could address these issues by prioritizing the most comprehensive or recent reviews or by weighting outcomes to account for overlap. Moreover, all the included studies utilized data from diagnostic imaging modalities such as MRI, CBCT, and radiography. A recent observational study demonstrated that ML algorithms based on clinical measurement parameters—such as mouth opening, pain levels, and oral parafunctions—achieved high accuracy, with the Bagging algorithm emerging as the most effective ML model. These findings indicate that ML‐based predictive models relying on relatively simple and easily accessible clinical data could assist less experienced general dentists in the early detection of TMD. Further research into the use of clinical parameters for TMD diagnosis could enhance early detection while reducing dependence on costly imaging techniques (Yıldız et al. 2024).

### 4.2 Future Implications for Research and Practice

Although AI has the potential to detect and diagnose TMJ disorders, the clinical significance of the results may be limited. The small number of studies indicates that AI models for TMJ diagnosis require further validation. Moreover, before AI models are implemented in clinical settings, the data used by these models must be thoroughly refined and evaluated by researchers and healthcare professionals to ensure their applicability and prevent unnecessary healthcare costs and potential adverse outcomes.

## 5 Conclusion

AI has the potential to deliver faster, more accurate, sensitive, and objective diagnoses of TMJ conditions, as evidenced by superior performance metrics. However, the effectiveness of AI depends on the models and datasets utilized. Therefore, before integrating AI‐based technologies into clinical practice, medical professionals must carefully assess their potential benefits for routine tasks. Additionally, researchers must thoroughly refine and evaluate the AI data before applying these models in clinical settings.

## Author Contributions

**Vini Mehta:** conceptualization, writing – review and editing, resources and supervision. **Snehasish Tripathy:** data acquisition, analysis and original manuscript writing. **Toufiq Noor:** writing – review and editing, resources and supervision. **Ankita Mathur:** writing – review and editing, resources and supervision.

## Ethics Statement

The authors have nothing to report.

## Consent

The authors have nothing to report.

## Conflicts of Interest

The authors declare no conflicts of interest.

## Supporting information

Supporting information.

## Data Availability

The data that supports the findings of this study are available in the Supporting material of this article.
